# Skeletal muscle mass adjusted by height correlated better with muscular functions than that adjusted by body weight in defining sarcopenia

**DOI:** 10.1038/srep19457

**Published:** 2016-01-20

**Authors:** Der-Sheng Han, Ke-Vin Chang, Chia-Ming Li, Yu-Hong Lin, Tung-Wei Kao, Keh-Sung Tsai, Tyng-Grey Wang, Wei-Shiung Yang

**Affiliations:** 1 Department of Physical Medicine and Rehabilitation, National Taiwan University Hospital BeiHu Branch, Taipei; 2 Department of Family Medicine, National Taiwan University Hospital BeiHu Branch, Taipei; 3 Department of Social Work, National Taiwan University Hospital BeiHu Branch, Taipei; 4 Department of Internal Medicine, National Taiwan University Hospital BeiHu Branch, Taipei; 5 Division of Geriatric Medicine, Department of Family and Community Medicine, Tri-Service General Hospital, National Defense Medical Center, Taipei; 6 Department of Internal Medicine, National Taiwan University Hospital, Taipei; 7 Department of Physical Medicine and Rehabilitation, National Taiwan University Hospital, Taipei; 8 Research Center for Developmental Biology and Regenerative Medicine, National Taiwan University, Taipei, Taiwan

## Abstract

Sarcopenia, characterized by low muscle mass and function, results in frailty, comorbidities and mortality. However, its prevalence varies according to the different criteria used in its diagnosis. This cross-sectional study investigated the difference in the number of sarcopenia cases recorded by two different measurement methods of low muscle mass to determine which measurement was better. We recruited 878 (54.2% female) individuals aged over 65 years and obtained their body composition and functional parameters. Low muscle mass was defined as two standard deviations below either the mean height-adjusted (hSMI) or weight-adjusted (wSMI) muscle mass of a young reference group. The prevalence of sarcopenia was 6.7% vs. 0.4% (male/female) by hSMI, and 4.0% vs. 10.7% (male/female) by wSMI. The κ coefficients for these two criteria were 0.39 vs. 0.03 (male/female), and 0.17 in all subjects. Serum myostatin levels correlated positively with gait speed (r = 0.142, p = 0.007) after adjustment for gender. hSMI correlated with grip strength, cardiopulmonary endurance, leg endurance, gait speed, and flexibility. wSMI correlated with grip strength, leg endurance, gait speed, and flexibility. Since hSMI correlated more closely with grip strength and more muscular functions, we recommend hSMI in the diagnosis of low muscle mass.

As patients age, one of the major geriatric syndromes is sarcopenia, which is the decrease of skeletal muscle mass and function. The impact of sarcopenia on the elderly is extensive, and includes muscle weakness, frailty, functional decline^1^, falling^2^, dependence, early institutionalization, and even mortality^3^,^4^. However, due to different diagnostic criteria, geographical locations, ethnicities, age, and measurement tools, the reported prevalence of sarcopenia varies greatly across different countries. In Finland, sarcopenia prevalence has been estimated to be as low as 0.9% in 70 to 80-year-old women as determined by dual energy X-ray absorptiometry (DXA)^5^. At the other extreme, Bahat *et al.* reported that the prevalence of sarcopenia, as determined by fat free mass measured by bio-impedance analysis (BIA), was over 85.4% in men older than 60 years of age in a nursing home in Turkey^6^.

The allocation of public health resources depends on the actual disease burden. In order to compare studies across the world, the European Working Group on Sarcopenia in Older People (EWGSOP) developed a practical clinical definition for sarcopenia in 2010^7^. This operational definition emphasizes the consideration of not only muscle mass but also muscle functions, that is grip strength and gait speed, and provides a platform for epidemiological and future therapeutic comparisons.

Different definitions of decreased skeletal muscle mass (SMM) were described in the EWGSOP criteria, which made inter-study comparisons difficult. Janssen *et al.* employed a BIA-derived skeletal muscle index (SMI), which was adjusted by body weight (wSMI), below two standard deviations (SDs) of young adult values as criteria in the analysis of a nationwide survey; this study found a relatively high prevalence of sarcopenia^8^. Baumgartner *et al.* employed DXA-derived SMI, which was adjusted by squared body height (hSMI), below two SDs of a young reference group as criteria; this study reported a lower prevalence and a high correlation with disability^9^. It is still debatable as to which of the two: wSMI or hSMI, is a better muscle-mass parameter to define sarcopenia.

In this study, we recruited apparently healthy elderly subjects in the community, and attempted to estimate the prevalence of sarcopenia in the northern urban areas of Taiwan using the EWGSOP criteria. We compared the aforementioned muscle mass parameters to muscle function parameters, and determined the cut-off value of skeletal muscle mass for sarcopenia. Additionally, we analyzed the association between body composition and serum myostatin levels.

## Methods

### Human subjects

This study was part of the Taiwan Fitness for Seniors Study (TAFITS), a prospective, observational cohort study. A total of 878 healthy volunteers (402 male, 476 female), over 65 years of age in 2012 in Taipei, Taiwan were recruited. The TAFITS was set up to examine the physical fitness and body composition of the citizens in urban areas in northern Taiwan. Patients with malignancy or active inflammatory diseases were excluded. The subjects were recruited from the Department of Health Check-up, National Taiwan University Hospital Bei-Hu Branch. One hundred and forty five young healthy volunteers (aged between 20 and 40 years old; 54 men, 91 women) were included as a reference group for body composition. All participants provided written informed consent, and the study was approved by the Research Ethical Committee of National Taiwan University Hospital (REC No.: 201303009RINC) conforming to the Declaration of Helsinki of the World Medical Association. The study was carried out in accordance with the nationally approved guidelines. Height was measured to the nearest 0.1 cm and weight to the nearest 0.1 kg on an electronic scale. Waist girth was measured midway between the last rib and the iliac crest using a soft tape while the patient was standing. Body mass index (BMI) was calculated as weight in kilograms divided by the square of the height in meters.

### Laboratory analysis

Ten milliliters of whole blood was withdrawn from the study participants in the morning after 12 hours of overnight fasting. The serum was isolated and immediately stored at −80 °C for subsequent assays. Serum myostatin levels were measured using competitive enzyme immunoassay (EIA) kits according to the manufacturer’s protocol (Immunodiagnostik AG, Bensheim, Germany). The test employed a polyclonal antibody against a full-length myostatin peptide. The sensitivity of the myostatin EIA was 270 pg/mL, and the intra- and inter-assay variabilities were less than 10% and 15%, respectively^10^.

### Body composition determination

Body composition measurements were conducted for all subjects using a multiple-frequency bioelectrical impedance analyzer (MFBIA) model InBody 720 (Biospace, Seoul, Korea). As one of the latest impedance analyzers, InBody 720 uses a tetra-polar 8-point tactile electrode system that measures the total and segmental impedance and phase angle of alternating electric current at six different frequencies (1, 5, 50, 250, 500, and 1000 kHz). Impedance measurements were made with the subject standing in an upright position, on foot electrodes on the platform of the instrument with bare feet, and gripping the two palm-and-thumb electrodes in each respective hand. The skin and the electrodes were pre-cleaned with the specific electrolyte tissue according to the manufacturer’s instructions. The subjects were instructed to empty their bladders before measurement. All body composition data were calculated using the instrument with the manufacturer’s proprietary software. The report provided the measured impedance at each frequency, as well as body weight, SMM, fat mass (FM), and BMI. MFBIA has been validated for the accuracy of body composition estimations in the elderly. The coefficient of variance of repeated measurements was 1.9%^11^,^12^.

### Measurement of health-related physical fitness

#### Gait speed

The usual gait speed was measured according to the criteria given by Lauretani *et al.*^13^. The subject was asked to walk at his/her usual speed for 7 meters. The time spent from 1.5 to 5.5 meters was recorded. The gait speed was calculated using the following formula:

Gait speed (m/s) = 4/time spent in seconds.

#### Grip strength

The maximal grip force of the dominant hand was measured with shoulder adducted, elbow flexed at 90°, and forearm in a neutral position to represent upper extremity muscle strength using an analog isometric dynamometer (Baseline® hydraulic hand dynamometer, Fabrication Enterprises Inc., Irvington, NY, USA). The highest value of three squeezes was used in the analysis^14^.

#### Leg endurance

Leg endurance was evaluated using a sit-to-stand test. Participants were asked to stand and sit repeatedly for 30 seconds with their arms folded across their chest, and the number of full strides was recorded^15^,^16^.

#### Cardiopulmonary endurance

Cardiopulmonary endurance was recorded as the number of full steps taken in two minutes. For each step, the participants were required to raise each knee to the midway point between the patella and iliac crest^15^,^17^.

#### Flexibility

Flexibility was measured by the chair sit-and-reach test. The subject sat at the front edge of a chair, extending one leg and placing the other foot flat on the floor. With hands on top of each other and arms outstretched, the subject reached as far forward as possible towards their toes. The score was documented as the distance between the tips of the middle fingers and the toes. A negative score indicated the failure of the finger tips to touch the toes, whereas a positive value demonstrated the capability to move the finger tips beyond the toes^15^,^17^.

#### Definition of sarcopenia

Sarcopenia was diagnosed using the criteria developed by the EWGSOP; both low muscle mass and low muscle function criteria were fulfilled. Low muscle function includes low strength or poor performance. According to the recommendations of the EWGSOP, low grip strength is considered to be 30 kg in men and 20 kg in women. Gait speed below 0.8 m/sec was regarded as poor muscular performance^7^.

Low muscle mass was defined using two criteria. The first was a hSMI of less than two SDs below the gender-specific young adult reference value: hSMI equal to SMM (kg) divided by the square of body height (m)^9^. The second was a wSMI of less than two SDs below the gender-specific mean value of the young reference group: wSMI equal to SMM (kg) divided by the body weight (kg)^8^. In this study, the cut-off values for low muscle mass were 7.40 kg/m^2^ (hSMI) and 35.7% (wSMI) for men and 5.14 kg/m^2^ (hSMI) and 30.7% (wSMI) for women.

Pre-sarcopenia was defined as low muscle mass only. Severe sarcopenia was diagnosed when low muscle mass, low strength and poor performance were all present.

#### Statistical analysis

Descriptive statistics was used to calculate the mean and SD for demographic, biochemical, and physical variables. We employed the independent t-test and the chi-square test to compare the differences in continuous and categorical variables, respectively, between genders. Correlation analysis was performed to find the Pearson’s partial correlation coefficients between SMM and the parameters for body composition and physical function. We used Cohen’s κ statistics to evaluate the agreement between different muscle mass criteria^18^. We specified sarcopenia as binary categories and estimated the gender-specific κ coefficients.

Binary logistic regression models were employed to estimate the odds ratios (ORs) for pre-sarcopenia. Pre-sarcopenia diagnosed by hSMI and wSMI was the dependent variable. Potential confounding variables including age, gender, BMI, and serum myostatin were chosen as independent variables. All analyses were performed using SPSS for Windows, Version 19.0 (SPSS Inc. Chicago, Illinois, USA), and a *p-*value < 0.05 was considered statistically significant.

## Results

**Figure 1:**
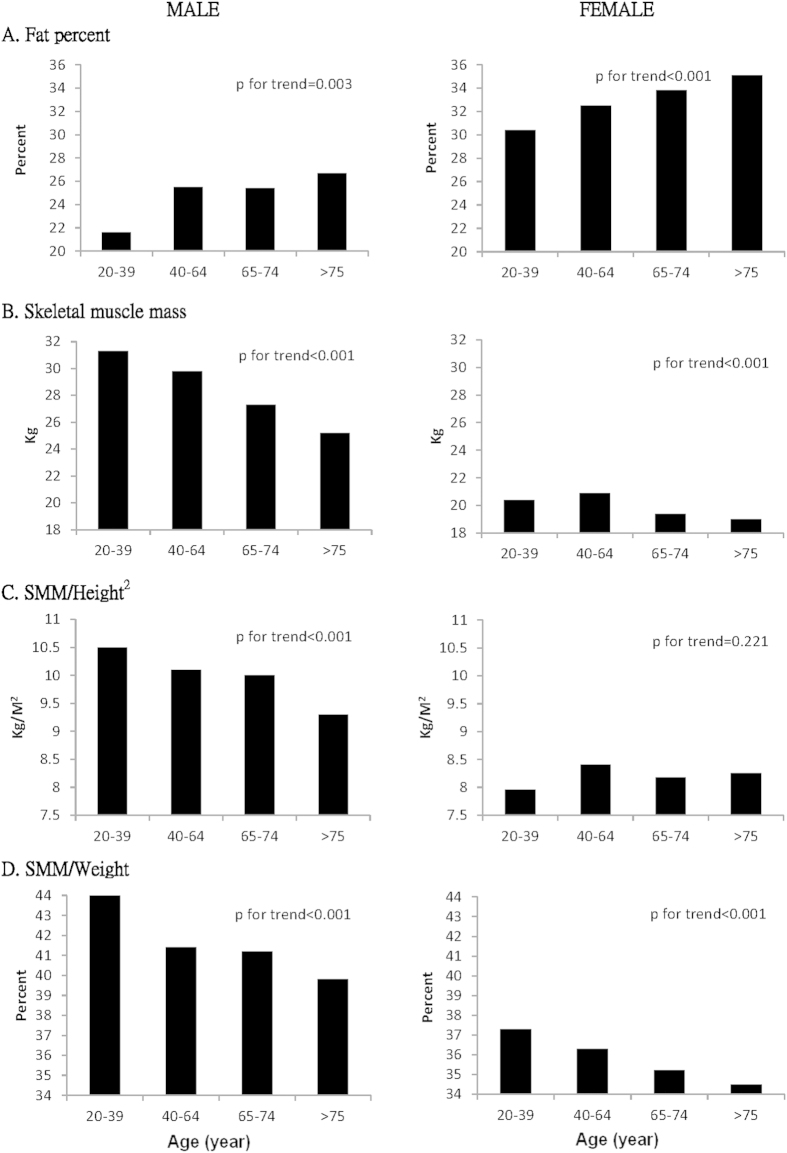
Trend analysis in fat and muscle mass in terms of age.

The characteristics of 878 community-dwelling elderly individuals are shown in Table 1. The average age was 72.7 ± 5.7 years, and there were no age differences between men and women. The male subjects had greater SMM, grip strength, leg endurance, gait speed, and higher levels of serum myostatin, while the female subjects had larger fat percentages, and flexibility. Body compositions, of both fat and muscle mass, showed different trends with age (Fig. 1). In aging males, the fat percentage increased and the SMM decreased, regardless adjustments for height or weight. The female subjects also showed a trend towards increased fat percentage and decreased SMM with aging. However, the declining trend was not significant when muscle mass was adjusted for height.

**Figure 2:**
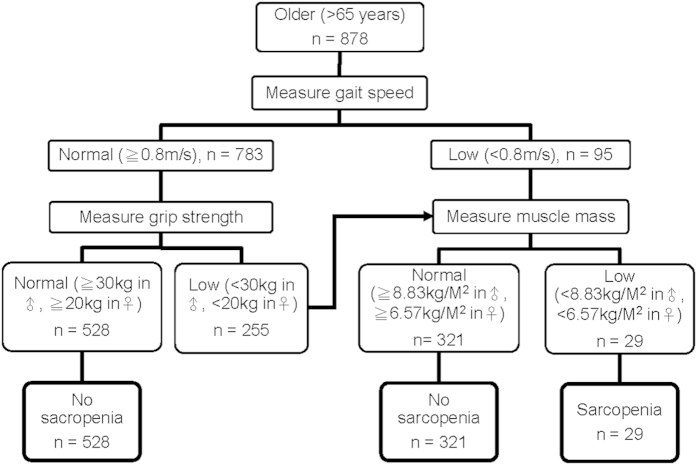
The EWGSOP algorithm for sarcopenia screening.

We employed both hSMI and wSMI to define low muscle mass. Based on the criteria of the EWGSOP, when defined by hSMI the prevalence of sarcopenia in our study was 6.7% in men and 0.4% in women (Fig. 2). When defined by wSMI, the prevalence of sarcopenia was 4.0% in men and 10.7% in women (Table 2). The Cohen’s κ coefficients between these two criteria were 0.39 in men, 0.03 in women, and 0.17 in total. Generally, the agreement between these two diagnostic criteria was low in female subjects and the total population, and fair in male subjects. According to EWGSOP’s recommendations, we also calculated the prevalence of pre-sarcopenia, and severe sarcopenia. Again, the prevalence of both pre-sarcopenia and severe sarcopenia was higher in men when defined by hSMI and higher in women when defined by wSMI (Table 2).

Since age-related loss in muscle tissue is primarily important due to its functional consequences, we correlated the muscle mass with the functional parameters after adjusting for age and gender. In Table 3, we show that hSMI correlated significantly with gait speed (r = 0.109, p = 0.001), grip strength (r = 0.171, p < 0.001), leg endurance (r = 0.126, p < 0.001), cardiopulmonary endurance (r = 0.102, p = 0.003), and flexibility (r = 0.105, p = 0.002). The wSMI correlated with gait speed (r = 0.098, p = 0.004), grip strength (r = 0.105, p = 0.002), leg endurance (r = 0.101, p = 0.003), and flexibility (r = 0.102, p = 0.003). Since hSMI correlated with more items and higher coefficients in muscular functions than wSMI, we concluded that hSMI was a better parameter for muscle mass.

Serum myostatin levels were higher in men than in women (9.7 ± 4.4 vs. 8.1 ± 4.1 ng/mL, p < 0.001) and correlated positively with gait speed (r = 0.142, p = 0.007) after adjustment for gender. However, myostatin levels were not related to SMM, including hSMI, and wSMI, after adjustment for age and gender.

We then employed a binary logistic regression model to identify the risk factors for pre-sarcopenia. We set pre-sarcopenia as the dependent variable, and age, gender, BMI, and serum myostatin levels as the independent variables. Serum myostatin levels (confidence interval [CI] of OR = 1.02–1.23, p = 0.019) and the male gender (CI of OR = 1.60–84.61, p = 0.015) were associated with an increased likelihood of pre-sarcopenia by hSMI. A high BMI was associated with a protective effect on pre-sarcopenia (CI of OR = 0.47–0.85, p = 0.003). When defined by wSMI, a high BMI (CI of OR = 1.45–1.97, p < 0.001) increased the risk of pre-sarcopenia.

## Discussion

In this study we demonstrated that the prevalence of sarcopenia in Taiwan differs according to the various muscle mass parameters used in its diagnosis. When defined by hSMI, the prevalence was 6.7% in men and 0.4% in women. When defined by wSMI, the prevalence was 4.0% and 10.7% in male and female subjects, respectively. The agreement between males and females was low. Male subjects had higher percentage of sarcopenia when defined by hSMI. In contrast, female subjects had higher percentage of sarcopenia when defined by wSMI. The hSMI correlated better than wSMI with muscular functions in the elderly, and should be the criteria in defining sarcopenia.

In this study, the prevalence of sarcopenia varied a lot within the same cohort when we employed different parameters to define low muscle mass. The agreement between the patient groups defined by hSMI and wSMI was low. We observed that men have a higher prevalence of sarcopenia when defined by hSMI and women have a higher prevalence when defined by wSMI. Similar results were reported by the Korean Longitudinal Study on Health and Aging and the study in China by Wen *et al.*^19^,^20^,^21^. The main reason for this is that SMM adjusted by height showed an insignificant trend associated with age in female (Fig. 1C). Possible explanations might be post-menopausal osteoporosis or a cohort effect^22^,^23^. The older females usually had shorter body heights due to osteoporosis. Additionally, young controls were recruited from a cohort different from the elderly population and might have had less physical activity, different dietary habits, and a more sedentary lifestyle^5^.

Based on the EWGSOP criteria, the prevalence of sarcopenia in Taiwanese people aged over 65 years ranged from 5.4% to 23.6% in men, and 2.5% to 18.6% in women^22^,^24^,^25^,^26^. Comparing these studies which focused on patients of the same ethnicity, we found the discordance came from different instruments, cut-off values, and urbanization of participants. In 2014, Chen *et al.* proposed a consensus from the Asian Working Group for Sarcopenia (AWGS) and suggested the instrument, cut-off value for the Asian countries^23^. Both criteria include low muscle mass plus low muscle strength and/or low physical performance. The main differences in the Asian criteria were an additional screening strategy for specific populations and lower cut-off values. Both BIA and DXA were permitted to determine the body composition. Since the BIA method under-estimated the lean mass when compared to DXA^7^, we set-up our own young reference control value for muscle mass. The MFBIA-derived muscle mass from the independent variable and brand-specific proprietary regression formula was shown to be valid and accurate in both young and old populations^11^,^27^. The AWGS suggested 7.0 kg/m^2^ in men and 5.7 kg/m^2^ in women as the cut-off values. Our reference value was 7.40 kg/m^2^ in men and 5.14 kg/m^2^ in women, which resulted in a larger difference between genders. We suggest that all studies employing BIA in muscle mass determination should set up their own young reference group to avoid discrepancy between equipment.

Sarcopenia is associated with functional limitations and places the elderly at risk for physical disabilities, including lower extremity strength, endurance, and flexibility^8^,^28^. An ideal index for skeletal muscle mass should be an risk factor independent of obesity for health-related outcomes^29^,^30^. hSMI was first used as an index of relative SMM by Baumgartner *et al.* and has since been employed in many studies^9^. Janssen *et al.* originally employed wSMI to define sarcopenia and reported that this parameter was independently associated with disability^8^. We proposed hSMI as the more suitable criterion when defining sarcopenia based on two points. First, hSMI correlated better than wSMI with grip strength. Second, wSMI showed high negative correlation with fat percentage. This is also supported by the AWGS consensus^23^.

Myostatin is a negative regulator of muscle growth and was proposed as a “brake” during muscle mass homeostasis. The expression of myostatin increases to limit the growth of muscle tissue during the process of muscular growth^31^,^32^. Since the ageing population had a smaller muscle mass, the serum myostatin also declines as there is no need to hit the “brake”. The serum myostatin levels were previously shown to have a negative correlation with age^31^,^33^, and in this study we also observed similar findings (r = −0.100, p = 0.055, adjusted for gender). However, when muscle mass further declined and fulfilled the criteria for sarcopenia, higher myostatin levels increased the risk of sarcopenia^34^,^35^. In this study, myostatin levels were not related to SMM in the simple correlation study, but myostatin did increase the risk for pre-sarcopenia defined by hSMI in the logistic regression model. This might be due to the fact that muscle mass is not only affected by the “accelerator-brake” factors but also by the metabolic environment in which the muscle resides, and exercise loading. Myostatin inhibition with a monoclonal antibody was shown to be a novel intervention in muscle-related disorders, including sarcopenia^36^,^37^. Two phase II trials focused on sarcopenia and recruited weak elderly subjects who were prone to falling. Interim results showed good tolerance and increased muscle mass in the treatment group^38^. Serum myostatin might be a serum marker for therapeutic outcome monitoring of sarcopenia.

Our study exhibited some limitations, one of which was selection bias. The individuals recruited could not represent the general population since they were all from the health check-up department of a district hospital. Similar to the “healthy worker effect”, this bias might underestimate the true prevalence of sarcopenia^39^. Population-based sampling can resolve this problem. Furthermore, the ideal criteria for diagnosing sarcopenia should present the outcome, including falling, institutionalization, or even mortality. However, current cross-sectional data could not support this concept. Future longitudinal experiments will provide more long-term outcomes and data on the cause-effect role of risk factors of sarcopenia.

In conclusion, our study revealed that the prevalence of sarcopenia in elderly people living in urban areas of northern Taiwan was relatively low compared to the prevalence rates reported in other cross-sectional studies. Our results indicated that hSMI was a better index for muscle mass than wSMI because the former displayed a closer correlation with grip strength. A further longitudinal study is needed to delineate the incidence, outcome, and effective therapy for sarcopenia.

## Additional Information

**How to cite this article**: Han, D.-S. *et al.* Skeletal muscle mass adjusted by height correlated better with muscular functions than that adjusted by body weight in defining sarcopenia. *Sci. Rep.* **6**, 19457; doi: 10.1038/srep19457 (2016).

## Figures and Tables

**Table 1:** Subject characteristics in demography, physical function, and biochemistry.

Variables	Male	Female	Total	*p*value
N	402	476	878	
Age (year)	73.7 ± 6.1	71.9 ± 5.2	72.7 ± 5.7	<0.001
Clinical parameters				
Body height (cm)	164 ± 6	153 ± 5	158 ± 8	<0.001
Body weight (kg)	65.4 ± 8.6	55.6 ± 8.0	60.0 ± 9.6	<0.001
BMI (kg/m^2^)	24.1 ± 2.8	23.6 ± 3.1	23.8 ± 3.0	0.021
Waist girth (cm)	86.3 ± 7.6	79.6 ± 7.4	82.9 ± 8.2	<0.001
Body composition				
Fat percentage (%)	26.0 ± 6.2	34.3 ± 6.3	30.5 ± 7.5	<0.001
Skeletal muscle mass (kg)	26.5 ± 3.8	19.3 ± 3.3	22.6 ± 5.0	<0.001
hSMI (kg/m^2^)	9.8 ± 1.2	8.2 ± 1.3	8.9 ± 1.5	<0.001
wSMI (%)	40.6 ± 4.5	35.0 ± 5.4	37.6 ± 5.7	<0.001
Physical function				
Gait speed (m/sec)	1.10 ± 0.22	1.05 ± 0.24	1.08 ± 0.23	0.001
Grip strength (kg)	32.8 ± 7.1	19.9 ± 4.7	25.8 ± 8.7	<0.001
Leg endurance (times)	15.4 ± 5.1	14.6 ± 4.7	15.0 ± 5.0	0.013
Cardiopulmonary endurance (times)	87.7 ± 20.1	88.1 ± 21.1	87.9 ± 20.6	0.795
Flexibility (cm)	−4.9 ± 13.1	3.7 ± 11.3	−0.2 ± 12.9	<0.001
Blood Chemistry				
Myostatin (ng/ml)	9.7 ± 4.4	8.1 ± 4.1	8.9 ± 4.3	<0.001

BMI: body mass index. hSMI: height-adjusted skeletal muscle index. wSMI: weight-adjusted skeletal muscle index.

**Table 2:** Prevalence of presarcopenia and sarcopenia based upon two definitions.

Definition Prevalence (%)	SMM/height^2^		SMM/body weight	
	Male (n = 402)	Female (n = 476)	Male (n = 402)	Female (n = 476)
Presarcopenia	10.4	0.4	8.2	19.1
Sarcopenia	6.7	0.4	4.0	10.7
Severe sarcopenia	1.2	0	0.5	3.6

SMM: skeletal muscle mass.

**Table 3:** Partial correlation coefficient of skeletal muscle mass (SMM), height-adjusted skeletal muscle mass index (hSMI), and weight-adjusted skeletal muscle mass index (wSMI) with parameters on body composition and physical function after adjusting for age and gender.

	SMM (kg)	hSMI (kg/M^2^)	wSMI (%)
Height (cm)	0.489 (<0.001)	0.017 (0.581)	0.122 (<0.001)
Weight (kg)	0.615 (<0.001)	0.464 (<0.001)	−0.372 (<0.001)
Body mass index (kg/M^2^)	0.393 (<0.001)	0.484 (<0.001)	−0.472 (<0.001)
Waist girth (cm)	0.285 (<0.001)	0.269 (<0.001)	−0.386 (<0.001)
Gait speed (m/sec)	0.124 (<0.001)	0.109 (0.001)	0.098 (0.004)
Grip strength (kg)	0.317 (<0.001)	0.171 (<0.001)	0.105 (0.002)
Leg endurance (times)	0.056 (0.100)	0.126 (<0.001)	0.101 (0.003)
Cardiopulmonary endurance (times)	0.068 (0.047)	0.102 (0.003)	0.045 (0.189)
Flexibility (cm)	0.051 (0.130)	0.105 (0.002)	0.102 (0.003)
Myostatin (ng/ml)	−0.086 (0.100)	−0.056 (0.285)	−0.011 (0.828)

Data are expressed as partial correlation coefficient (p-value).
